# Genomic prediction for growth using a low-density SNP panel in dromedary camels

**DOI:** 10.1038/s41598-021-87296-7

**Published:** 2021-04-07

**Authors:** Morteza Bitaraf Sani, Javad Zare Harofte, Mohammad Hossein Banabazi, Saeid Esmaeilkhanian, Ali Shafei Naderi, Nader Salim, Abbas Teimoori, Ahmad Bitaraf, Mohammad Zadehrahmani, Pamela Anna Burger, Vincenzo Landi, Mohammad Silawi, Afsaneh Taghipour Sheshdeh, Mohammad Ali Faghihi

**Affiliations:** 1 Animal Science Research Department, Yazd Agricultural and Natural Resources Research and Education Center, Agricultural Research, Education & Extension Organization (AREEO), 8915813155 Yazd, Iran; 2 Department of Biotechnology, Animal Science Research Institute of IRAN (ASRI), Agricultural Research, Education & Extension Organization (AREEO), 3146618361 Karaj, Iran; 3 Department of Biotechnology, Animal Science Research Institute of IRAN (ASRI), Agricultural Research, Education and Extension Organization (AREEO), 3146618361 Karaj, Iran; 4 Organization of Agriculture - Jahad -Yazd, Ministry of Agriculture-Jahad, 8916713449 Yazd, Iran; 5 Yazd Dar Al-Elm Higher Education Institute, Yazd, Iran; 6 Research Institute of Wildlife Ecology, Vetmeduni Vienna, 1160 Vienna, Austria; 7 Departement of Veterinary Medicine, Università Di Bari “Aldo Moro”, Bari, Italy; 8 Persian BayanGene Research and Training Center, 7134767617 Shiraz, Iran; 9 Center for Therapeutic Innovation and Department of Psychiatry and Behavioral Sciences, University of Miami, Miami, FL 33136 USA

**Keywords:** Genomic analysis, High-throughput screening, Sequencing

## Abstract

For thousands of years, camels have produced meat, milk, and fiber in harsh desert conditions. For a sustainable development to provide protein resources from desert areas, it is necessary to pay attention to genetic improvement in camel breeding. By using genotyping-by-sequencing (GBS) method we produced over 14,500 genome wide markers to conduct a genome- wide association study (GWAS) for investigating the birth weight, daily gain, and body weight of 96 dromedaries in the Iranian central desert. A total of 99 SNPs were associated with birth weight, daily gain, and body weight (p-value < 0.002). Genomic breeding values (GEBVs) were estimated with the BGLR package using (i) all 14,522 SNPs and (ii) the 99 SNPs by GWAS. Twenty-eight SNPs were associated with birth weight, daily gain, and body weight (p-value < 0.001). Annotation of the genomic region (s) within ± 100 kb of the associated SNPs facilitated prediction of 36 candidate genes. The accuracy of GEBVs was more than 0.65 based on all 14,522 SNPs, but the regression coefficients for birth weight, daily gain, and body weight were 0.39, 0.20, and 0.23, respectively. Because of low sample size, the GEBVs were predicted using the associated SNPs from GWAS. The accuracy of GEBVs based on the 99 associated SNPs was 0.62, 0.82, and 0.57 for birth weight, daily gain, and body weight. This report is the first GWAS using GBS on dromedary camels and identifies markers associated with growth traits that could help to plan breeding program to genetic improvement. Further researches using larger sample size and collaboration of the camel farmers and more profound understanding will permit verification of the associated SNPs identified in this project. The preliminary results of study show that genomic selection could be the appropriate way to genetic improvement of body weight in dromedary camels, which is challenging due to a long generation interval, seasonal reproduction, and lack of records and pedigrees.

## Introduction

For thousands of years' camels have produced meat, milk, and fiber in harsh desert conditions. There are 35 million camels globally (FAO, 2019), 95% of which are dromedaries^1^. The innate characteristics of adaptability and sustainability of the productions can be in antagonism as already proven for example in the bovine species^2^. Thus, the development of a modern genetic improvement program for the productivity of the dromedary should be accompanied by a profound understanding of its genome and the mechanisms of inheritance of the characters of interest^3^. The ability to blend together the adaptability to hot climates and its innate rusticity with an efficient production capacity would make this animal an excellent alternative in marginal environments^3^. In local population, the lack of phenotypic records and pedigrees, small herd size and missing connectedness, and genetic evaluations are the main limitations^4^. Genomic approaches can be beneficial to reduce the impact of these problematic^5^.

Next-generation sequencing platforms have prepared suitable approaches for genome wide association studies and genomic selection at the whole-genome level^6^. By using genotyping-by-sequencing (GBS) method can be produced many genome wide markers, which is a that supports GWAS^7,8^. GBS has been widely used in plant and animal breeding for genome-wide association analysis, genomic diversity studies, and genomic selection^9^. The availability of reference genome assemblies coupled with GBS enables us to explore in greater detail of dromedary populations and to identify genetic associations with different phenotypic traits^10^. Genome-wide association studies (GWAS) are used to screen the whole genome for target genes that correlate with phenotypic traits, using SNPs and an important method for identifying candidate genes for important economic traits in livestock^11^. GWAS have a greater capability than QTL mapping to detect causal SNPs in a smaller genetic range^12^. In recent years, many genes and molecular markers, regulate important traits, were identified using GWAS in livestock animals like pigs, cattle, sheep, and chickens^13,14^. Despite its unique potential and increased contribution to food security, comparatively less attention has been paid to camels compared to other livestock species^3^.

Body growth is an economical important trait in dromedaries. The birth and weaning weight, gain per day, and body weights at different ages are used to reflect the growth and development. Growth in weight is a heritable trait and an important index of selection^17^. Although meat production and its functionality are strategic topics in camel breeding, which is reflected by the increasing interest of stakeholders and consumers in camel products, few studies have been produced in this field.

In Iran, there are about 140,000 dromedary camels (FAO, 2019) that are divided into four basic types: meat type, milk type, dual purpose and riding camels^18^. Camels in the central desert of Iran usually belong to the meat type with a large and heavy muscular head, short neck, large hump, wide posterior parts and firm body^19^. The camels are kept by the villagers located around the desert. The camels are gathered usually in spring. While the young camels are sold the remaining herds are returned to the desert. During the summer, the camels need more water, so they return to the villages every day. The size of herds may vary from 4–5 up to 100–150 animals.

The present study has been designed to gain first knowledge on body weight traits at different age in the Iranian dromedary population, and to understand their underlying genomic characteristics. We collected a large number of phenotypic measurements in 96 dromedaries in the Central Desert of Iran as well as genotypes by GBS approach. We applied GWAS to identify candidate genes for growth related traits and estimated GEBVs for selected SNPs. This study is a first step towards systematic genomic breeding and selection in dromedaries for the benefit of local communities depending on camels as resource for income and food.

## Materials and methods

### Ethics statement

All of the animal procedures were performed in strict accordance with the guidelines and regulations proposed by the Animal Science Research Institute of Iran. All the animal experiments were approved by ethics committee of the Animal Science Research Institute of Iran under the number ASRI-34–64-1357–005-970,180. Blood samples were collected during qualified veterinary treatment within the framework of governmental programs aimed at the animal identification, monitoring of health, and parentage confirmation of the dromedary populations included in our study. No other kind of tissue was used in this study.

### Animals and sample collection

Yazd province with the area of 129,285 km^2^ (49,917 sq mi) is situated at an oasis where the Dasht-e Kavir desert and the Dasht-e Lut desert meet and located in 31° 53′ 41.28″ N, 54° 21′ 25.2″ E (31.8948, 54.357). Data on 51 herds of dromedaries were collected in 2018. The herd size mean was 89.90, range from 11 to 400 heads among 4279 camels, 16% were younger than one year, and named Hashi, while 12% were older than ten years. The proportion of females (Arvaneh), males (Lok), male calves (Hashi), and female calves (Hashi) were 76%, 9%, 6%, and 9%, respectively. Among pregnant camels, 49% were older than ten years. The ratio of pregnant camels to all female camels was 46%. A total of 964 calving was registered between January to May 2018, distributed over 22% in January, 28% in February, 27% in March, 15% in April, and 8% in May. Generation intervals in females and males were estimated at 7.84 and 5.91 years, respectively. Among 256 male calves, we recorded 96 samples from 5 regions including: Bafgh (n = 41) Bahabad (n = 8), Khatam (n = 17), Mehriz (n = 8), and Ardakan (n = 22). Characteristics of the sampled herds and the rangeland plants are presented in Table 1.

**Table 1:** Characteristics of sampling herds and the rangeland plants.

Region	Herd size	Sampling site	n samples	The rangeland plants
Bahabad	400	31°52′29.6"N 56°01′14.7"E	8	*Seidlitzia Rosmarinus, Artemisia spp, Salsola yazdiana, Tamarix tetragyna, Alhagi camelorum, Calligonum comosum, Zygophyllum spp*
Khatam	112	30°28′42.4"N 54°12′36.7"E	17	*Seidlitzia rosmarinus-Haloxylon ammodendron, Artemisia sieberi-Seidlitzia Rosmarinus, Tamarix tetragyna, Alhagi camelorum, Salsola yazdiana-Calligonum polygonoides, Zygophyllum atriplicoides*
Ardakan	450	32°31′40.8"N 55°14′32.9"E	22	*Salsola yazdiana, Haloxylon ammodendron, Artemisia sieberi , Zygophyllum spp*
Mehriz	320	31°35′14.4"N 54°25′44.4"E	8	*Haloxylon ammodendron-Zygophyllum atriplicoides, Artemisia sieberi-Salsola arbuscula,*
Bafgh*				*Zygophyllum spp, Seidlitzia Rosmarinus, Artemisia sieberi, Zygophyllum spp, Salsola yazdiana*
Herd1	117	31°37′05.0"N 55°24′26.9"E	23	
Herd2	100		18	

*Two herds in Bafgh were recorded (1: local herd and 2: National Research and Development Station on Dromedary Camel herd).

### Phenotypic measurements

**Figure 1:**
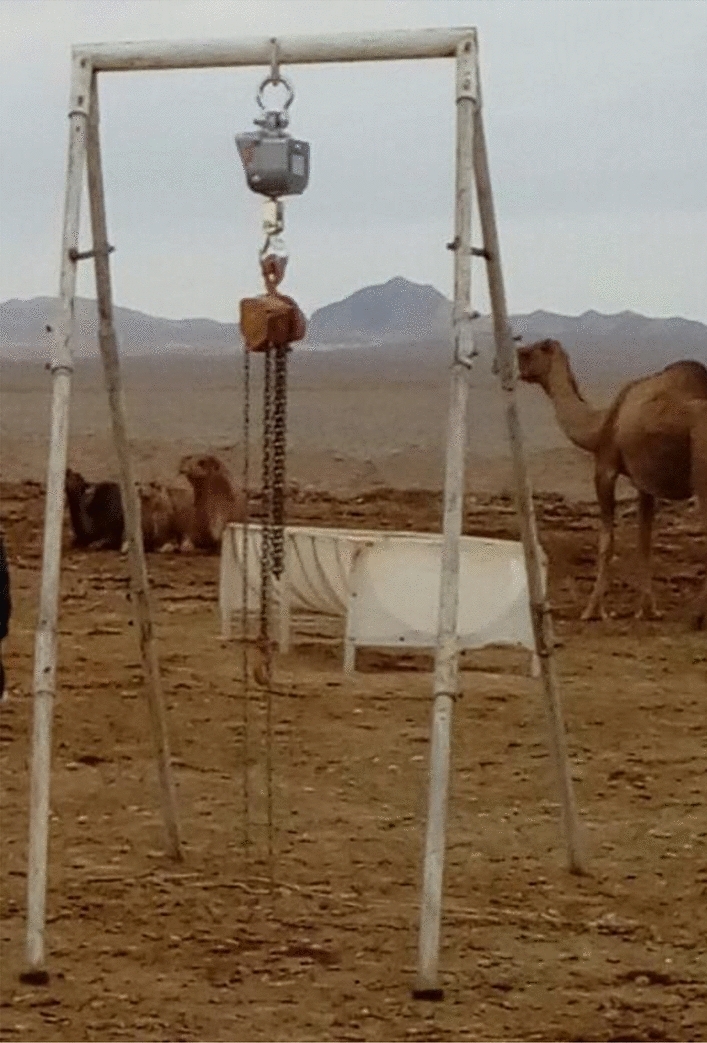
The portable weighting scale (Company: Fuzhou Kejie Intelligent Technology Co.,Ltd. model: OCS-XZ-2, 2000 Weigh Capacity (Kg), Accuracy Class III, + /- 1 division (least count).This were made from 13 pieces of iron, hanging digital scale 2000 kg, and one chain crane.

Data were recorded at the morning before grazing on the pasture. Camels were kept in closed area at night, which is called Garch. The animal identification was inferred via three-digit ear tags. Due to large distance in remote regions and transport difficulties, we constructed a portable weighting scale, consisting of 13 pieces of iron, a digital scale for 2000 kg, and one chain crane (Fig. 1). The meta data collected for any calf included: ID number, characteristics of owner, geographical region, recording date, birth date, parental names, and body weight. We recording intervals were approximately three months, with the first record starting in the calving season, the second during the summer, and the third at weaning season at the beginning of autumn. We collected 252 body weight records from 96 calves in different times during 2018. The 18 calves belong to National Research and Development Station on Dromedary Camel (Bafgh), measured in 8 -times, the others were recorded 2 or 3-times including: Bafgh (n = 164), Bahabad (n = 8), Khatam (n = 26), Mehriz (n = 9), Ardakan (45).

For adjusting the body weight trait, the growth trend was estimated using linear regression model (Eq. 1). Analysis of covariance (ANCOVA) of body weight was did among five sampling regions using SPSS v.22 software^20^. The mean of differences was compared using LSD test. Daily weight gain was calculated by two or three-times records.

1 $${y}_{ij}=\mu +{\tau }_{i}+\beta \left({x}_{ij}-\stackrel{-}{x} \right)+{\epsilon }_{ij}$$

${y}_{ij}$: body weight; $\mu :$ Effect of mean; ${\tau }_{i}$: sampling regions includes: (Bafgh, Bahabad, Khatam, Ardakan, and Mehriz); $\beta :$ The regression coefficient; ${x}_{ij}$: Age of recording; $\stackrel{-}{x}$: average of recording Ages.

### DNA extraction, SNP discovery and genotyping

Blood samples were collected from the jugular vein in EDTA tubes during routine veterinary treatment on the pasture. The genomic DNA was extracted using the modified salting-out method^21^ and, after elution, was quantified using spectrophotometry and checked for quality on a 1% agarose gel. Finally, DNA samples were adjusted to a concentration of 50 ng/µl for subsequent steps.

The samples were genotyped-by-sequencing using two restriction enzymes combination, EcoR1 and HinF1 (New England Biolabs, Ipswich, MA, USA), and paired-end (150 bp) sequencing (10 X) on the Illumina HiSeq 2000 platform by Persian Bayangene Research and Training Center (Shiraz, Iran).

The sequence reads were mapped to the dromedary reference genome assembly on chromosome level (GCA_000803125.3[1];) by using the BWA-MEM algorithm of Burrows–Wheeler Aligner (BWA)^22^;. PCR duplicates were detected by utilizing Picard tools and disregarded in downstream analyses both by GATK^23^ and SAMtools^24^. SNPs were called across the GBS data using GATK.

### Population structure andd genetic diversity

A quality control (QC) steps, and genome-wide diversity (observed and expected heterozygosity), as well as admixture analyses were performed using TASSEL V5.023. Variants with a minor allele frequency (MAF) below 0.05 and call rate below 0.95 were removed. Of the 41,897 SNPs, 256 markers were monomorphic, and 27,375 markers were deleted because of MAF < 0.05. The final data set consisted of 14,522 SNPs and 96 individuals. To investigate population structure, we used vcfR package^25^ in R for data manipulation and quality control as for producing input file objects for the other analysis, after that using ape and poppr package^26^ we carried out K-means clustering and discriminant analysis of principal components (DAPC), while all graphics were produced by means of RColorBrewer^27^.

### Linkage disequilibrium analysis (LD) and SNP-based haplotype blocks estimating

TASSEL 5.0^28^ was used to calculate the linkage disequilibrium (LD) (r^2^) for all pairwise loci. Haplotype blocks (HAP) were constructed in Haploview^29^.

### Genome-wide association studies and candidate genes prediction

The association between the SNPs and the traits were tested using mixed linear models with PCA and kinship matrix in TASSEL software^28^. The regions of this study and age at weighting date were used as a fixed and covariate effect, respectively.

The statistical analysis model, the MLM-PCA + K analysis, was expressed as:

$$y=\alpha X+\beta P+UZ+e$$

where y was phenotype value; α was the vector of SNP effects; β was vector of population structure effects based on PCA; u was vector of kinship background effects; e was vector of residual effects; X, P, Z were incidence matrix relating the individuals to fixed marker effects α, fixed principal component (PC) effects β, random group effects u, respectively. The suggestive significant Bonferroni P-value thresholds were set (−log p value > 3.9) using the GEC software tool^30^. The associated SNPs (−Log p value > 3) was traced in NCBI and the candidate genes were detected by blasting to the dromedary camel’s genome (GCA_000803125.3). We considered genes associated with the respective SNPs, if they were located either within the exon/ intron of a gene or within a flanking region of 100 kb up- and downstream.

### Bayesian genomic prediction

The estimation of Genomic Breeding values (GEBVs) was performed with the BGLR package including BRR, Bayes A, B, and C approach (nIter = 100 k, Burn In = 5 k)^31^. two sets of SNPs were used to predict GEBV: (1) all 14,522 SNPs and (2) the 99 associated SNPs (-Log p value ≥ 2.5 from GWAS). The prediction accuracy was estimated using the average Pearson’s correlation (r) and regression (b) coefficient between the GEBVs and observed values^32–34^. The replicated training—testing approach (10 replications) was used for evaluation of the models. We also used 3:1 size ratio of training set and validation set randomly selected from the 96 camels, which is a three-folds cross-validation, and repeated 100 times for evaluation of models by the 99 associated SNPs^35,36^.

## Result

### Phenotypic statistics of body weight traits

**Figure 2:**
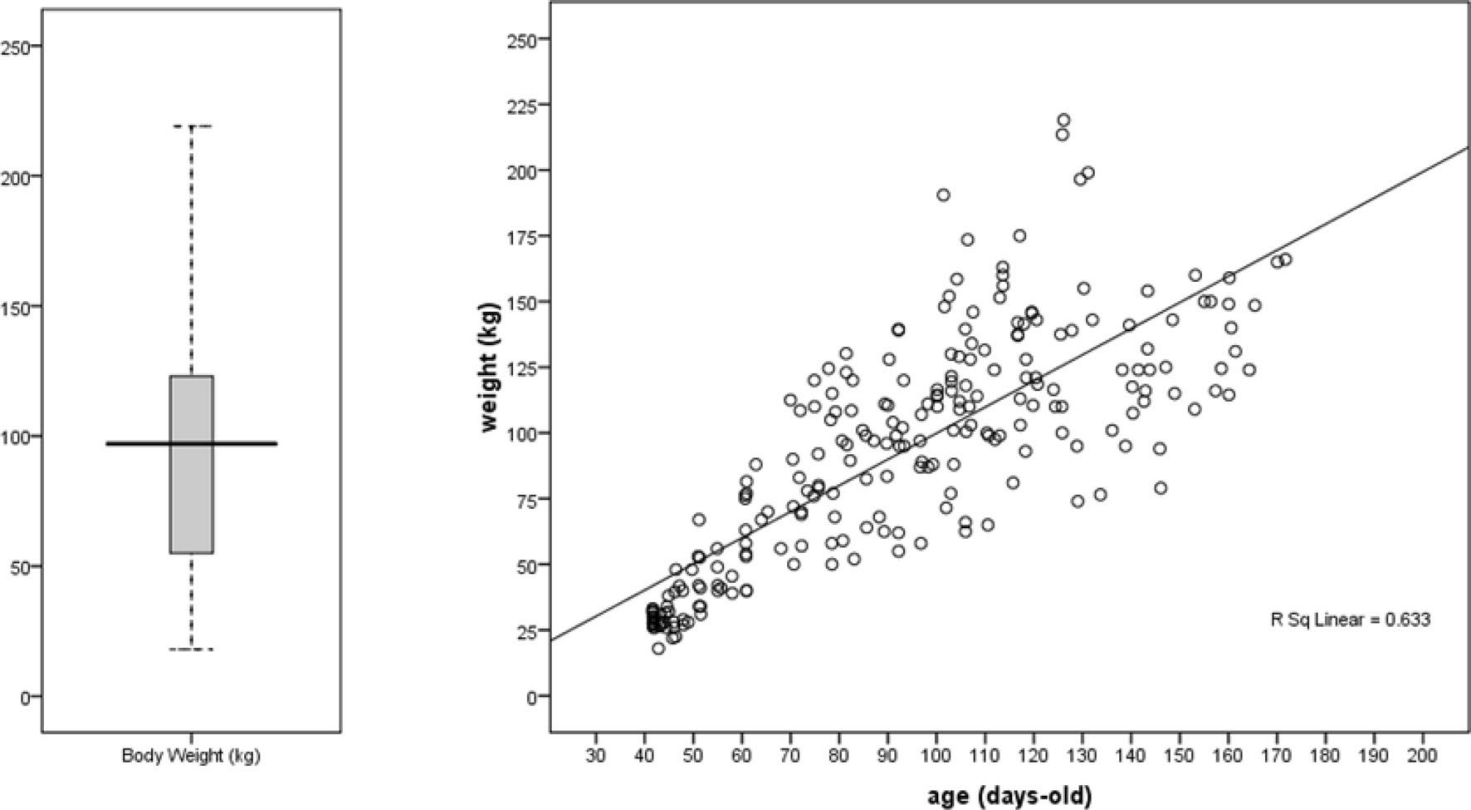
Box plot of 255 body weight records (left plot) and growth trend (right plot) of 96 dromedary camels in five regions of the central desert of Iran.

The distribution of 252 body weight records is visualized in Fig. 2. The growth trend of data suggested linear relationship between age and body weight (R_adj_^2^ = 0.63). Analysis of covariance for body weight records showed significant (p < 0.05) differences among camels from five sampled regions (Table 2). The body weight of camels in Ardakan was higher than the others (except Mehriz, because camels in addition to grazing on the pasture were fed by hand. The camels of Bafgh and Bahabad didn’t have significant differences. It is necessary to adjust the sampling region effect in GWAS and genomic selection.

**Table 2:** ANCOVA of body weight (kg) among five regions of the central desert of Iran.

Region	Mean*	SE	Confidence interval (95%)	N records	R_adj_^2^
Bafgh	82.90	1.68	79.58–86.22	156	0.77
Bahabad	82.57	8.12	66.55–98.58	7	
Khatam	94.07	4.12	85.94–102.20	26	
Ardakan	125.17	3.30	118.65–131.69	40	
Mehriz	115.96	7.42	101.33–130.59	8	

*Age of recording included as covariate and body weight are evaluated at the Age = 155.98 days-old.

**Figure 3:**
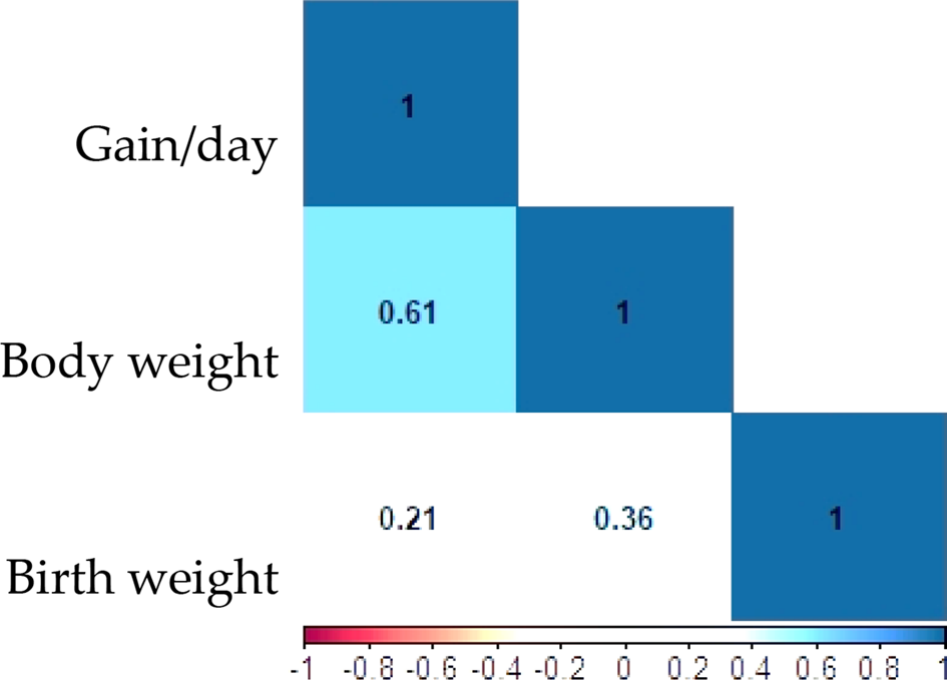
Correlation coefficients among birth weight, daily gain, and body weight in dromedary camels.

The adjusted birth weight, daily weight gains and body weight of the 96 camels from five regions of the Iranian central desert are shown in Table 2. The descriptive statistics including the mean, standard error (SE), coefficient of variation (CV) are presented in Table 3. The Pearson correction between daily gain with body weight (r = 0.63) was more than birth weight (r = 0.21). Also, the birth and body weight were correlated (r = 0.36) (Fig. 3).

**Table 3:** The descriptive statistics of body weight traits of dromedary camels.

Trait	Mean	SE	CV
Birth Weight (Kg)	28.95	0.93	31%
Daily gain (gr)	511.38	12.25	23.6%
Body Weight (kg) (age as covariate): From the birth date to 272 days age	90.08	44	48%

## Summary of genotyping data

**Figure 4:**
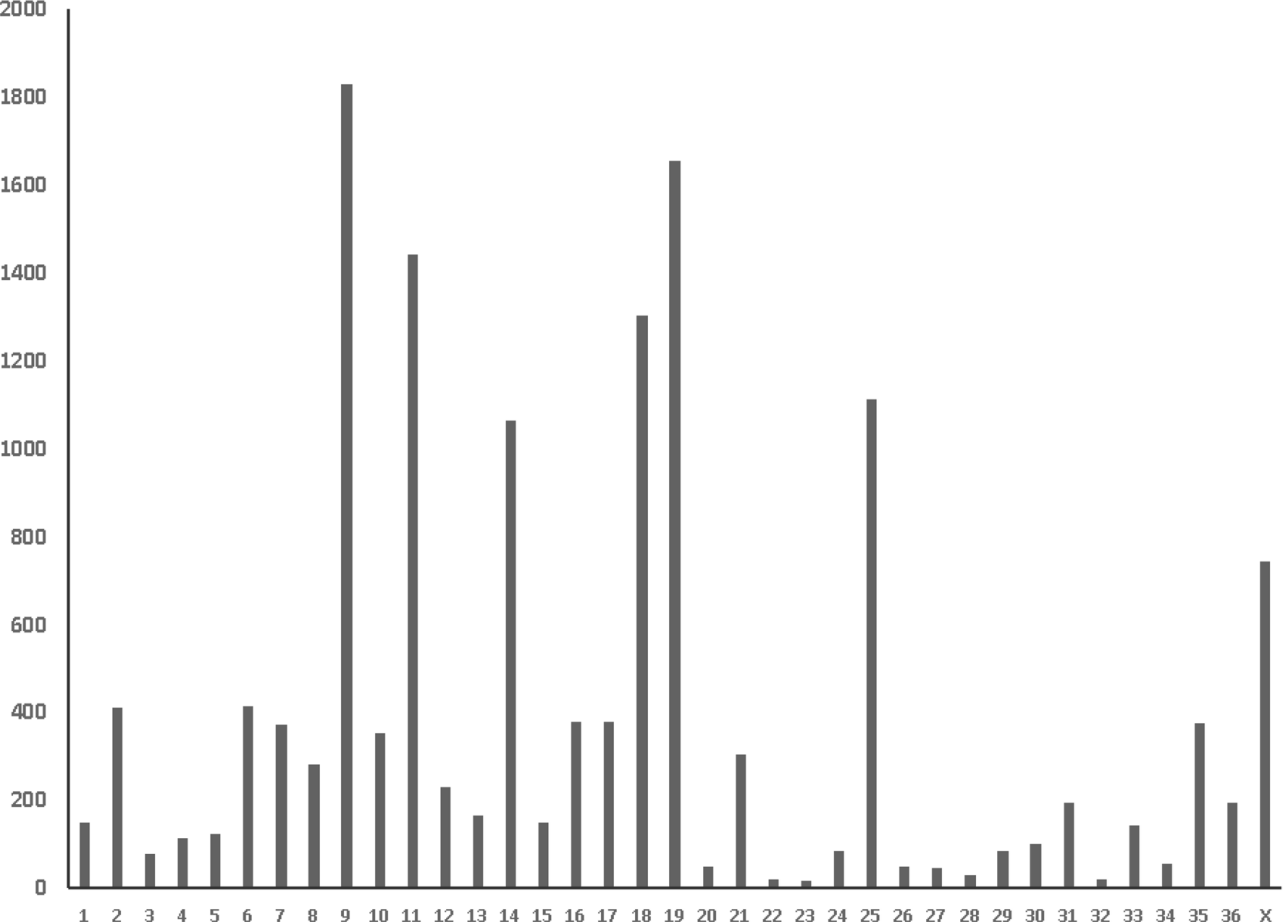
Number of SNPs/Chromosome.

**Figure 5:**
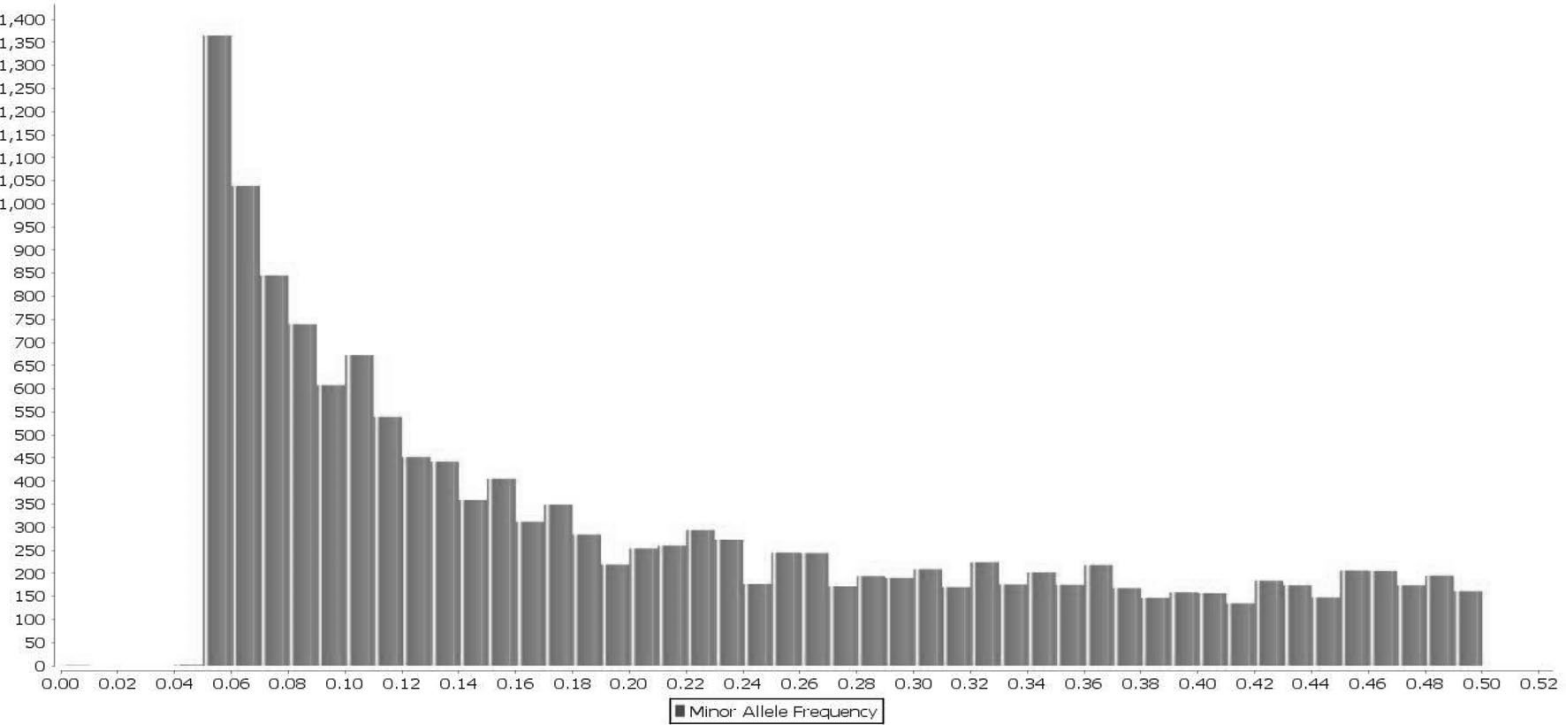
The Minor Allele Frequency distribution of 14,522 SNPs.

A total of 14,522 SNPs resulted after filtering and were used for final analysis. The largest number of SNPs was identified on chromosome 9 (n = 1829) followed by chromosome 19 (n = 1655), and the smallest number of SNPs was found on chromosomes 22 (n = 20) and chromosomes 23 (n = 16) (Fig. 4). The average MAF of all SNPs was 0.19, after QC (MAF > 0.05) (Fig. 5). Average observed heterozygosity was 0.25 ± 0.03.

### Genome wide association study

**Figure 6:**
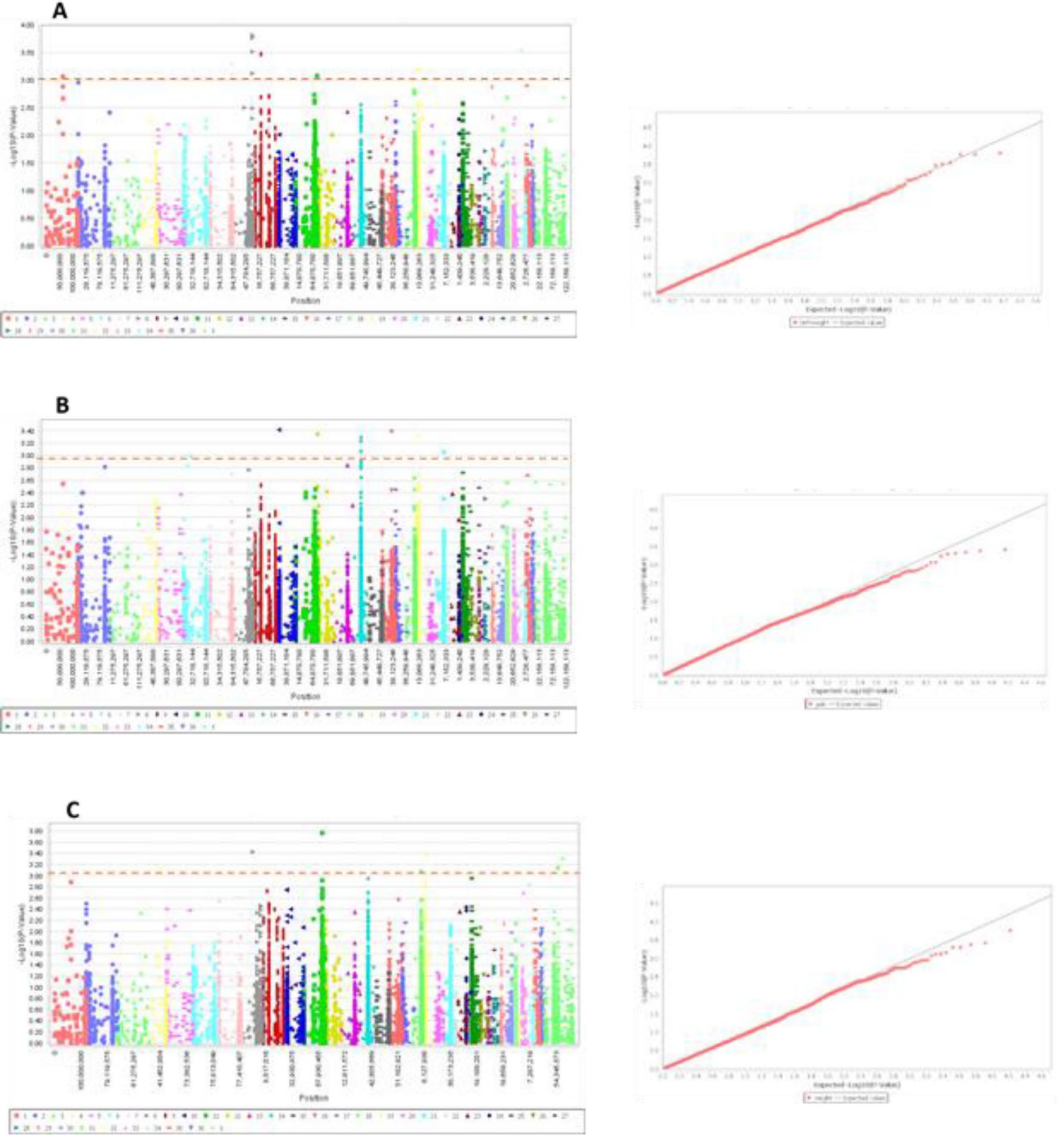
Manhattan plots and q-q plots of birth weight, daily gain, and body weight traits for Dromedary Camels. (**A**) birth weight; (**B**) daily gain; (**C**) body weight. The dotted horizontal line represents the set significant threshold (–log_10_ p value = 3). Red dots in the q–q plots represent the -log p-value of the entire study and the grey line represents the expected values under the null hypothesis of no association.

The MLM-PCA + K statistical model considering the covariates composed of population structure and kinship matrix was used for GWAS to prevent false positivity. In GWAS, the p value should be less than Bonferroni correction by using α/Me, where α = 0.05 and Me = effective number of SNPs. After applying a Bonferroni correction (1.2 × 10^–4^), no SNPs correlated with the growth traits (Fig. 6). However, this was expected given the limited number of samples used in our study (n = 96). A number of 28 SNPs were found to be associated with birth weight, daily gain, and body weight of dromedaries (p value < 0.001) (Table 4). For birth weight, 12 correlated SNPs (p value < 0.001) were detected on the chromosomes 7, 8, 9, 11, 19, and 34 were annotated to 9 genes (Table 4). For daily gain, 7 correlated SNPs (p value < 0.001) on the chromosomes 10, 16, 12, 19, and 14, were annotated to 11 genes (Table 4). For body weight, 9 correlated SNPs (p value < 0.001) on the chromosomes 11, 8, 19, X, 14, and 18 were annotated to 16 genes (Table 4). The most significant associated SNP with birth weight, daily gain, body weight, was located on chromosome 8, 10, and 11, respectively (-log p value = 3.81, 3.41, and 3.76, respectively). Out of the 36 genes potentially associated with peak SNPs [the genes listed in Table 4], two genes harboured the SNPs in their exon/ intron regions. Another 11 genes were detected in flanking regions of less than 30 kb up- and downstream of the respective SNP. Four genes were identified in 30–50 kb regions and 12 genes lay 50–100 kb up- and downstream of the potentially associated SNP.

**Table 4:** Genome-wide association studies (GWAS)-identified significant single-nucleotide polymorphisms (SNPs) (− log p value > 3), associated traits, and candidate genes.

Trait	Chromosome	pos	MAF	−log_10_ (p-Value)	Candidate gene
Birth weight	7	82,483,685	0.09	3.29	*ACTR3B, RPL32, XRCC2*
	7	82,483,675	0.08	3.10	
	8	72,594,633	0.11	3.81	*SERAC1*
	8	72,594,548	0.09	3.77	
	8	72,594,549	0.09	3.77	
	8	72,594,593	0.13	3.51	
	8	72,594,639	0.08	3.12	
	9	22,550,930	0.15	3.47	*TBX15*
	11	81,210,420	0.06	3.07	*mRNA-hypothetical protein and KAB1271495.1*
	19	10,237,661	0.05	3.21	*RNF114, IFNL1, SPATA2, SNAI1*
	19	10,237,641	0.05	3.18	
	34	6,774,561	0.06	3.54	UNKHOWN
Gain/day	10	13,892,353	0.06	3.41	UNKHOWN
	16	34,483,240	0.39	3.38	*EFCAB5, NSRP1, Slc6a4*
	12	5711	0.02	3.34	*ITGA7, OR6C2*
	19	9,631,630	0.17	3.32	*RIPOR3, PTPN1, PARD6B, BCAS4, MOCS3*
	14	31,371,259	0.11	3.29	*TRAPPC9*
	14	30,865,065	0.23	3.23	
	14	30,854,110	0.37	3.07	
Body weight	11	72,356,401	0.30	3.76	*EMX2, mRNA-hypothetical protein and KAB1271709.1*
	8	43,104,525	0.30	3.42	*FAM184A, MCM9, ASF1*
	19	10,894,226	0.34	3.38	*CSE1L, ARFGEF2*
	X	78,141,509	0.43	3.30	*TSR2, WNK3*
	X	78,141,514	0.43	3.30	*TSR2, WNK3*
	X	60,452,363	0.08	3.16	*LPAR4, RTL3, PEG10*
	X	62,116,802	0.06	3.12	*PBDC1, TRAPPC13*
	4	40,844,257	0.16	3.13	UNKNOWN
	18	29,958,631	0.06	3.08	*DEXI, TSPYL4, CIITA*

**Figure 7:**
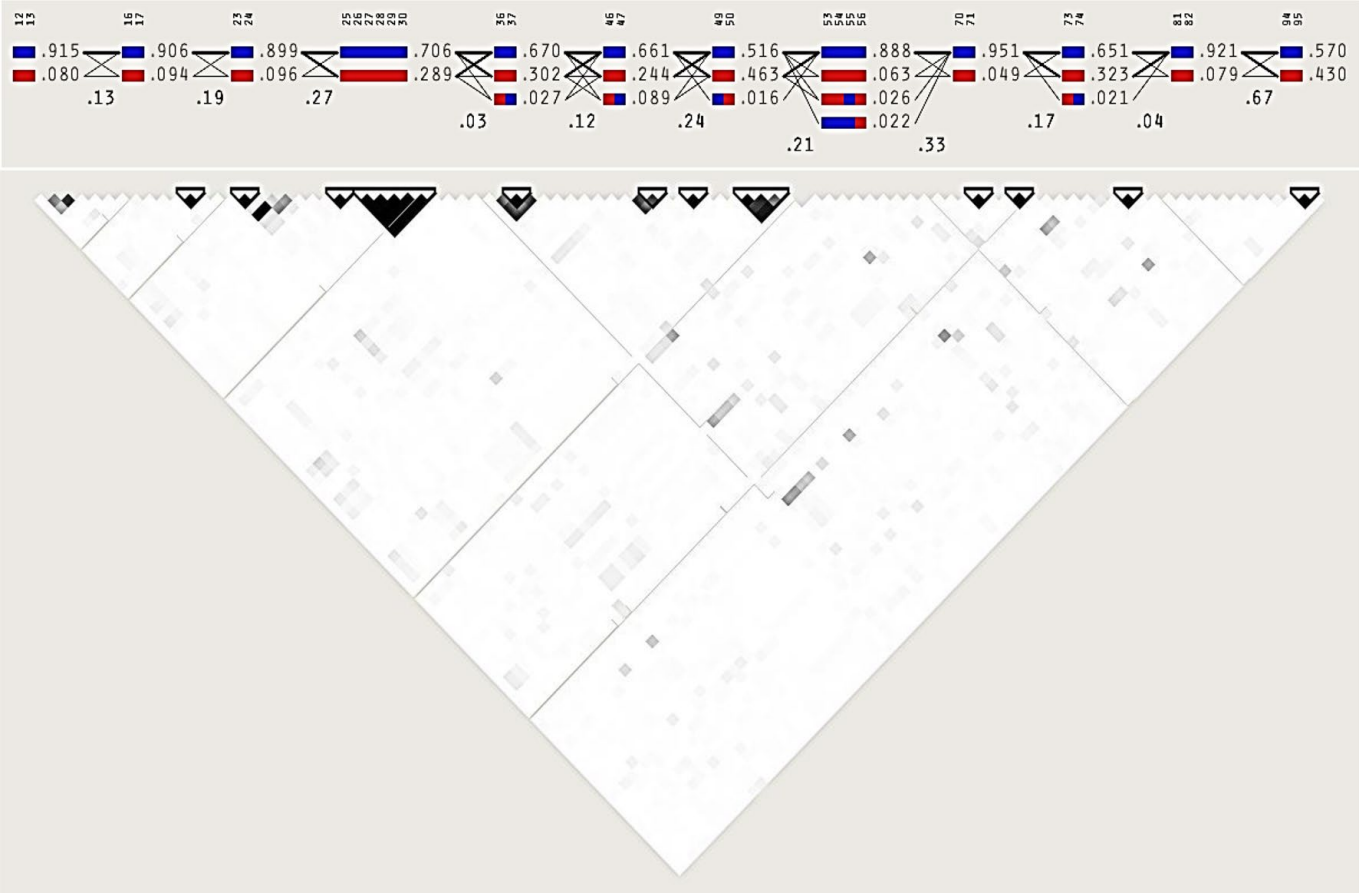
the Haplotype Blocks of the 99 associated SNPs with birth weight, daily gain, and body weight.

A total of 99 SNPs were associated with the three traits (birth weight, daily gain, and body weight) at p value < 0.002 (Table 5). Twelve haplotype blocks and 80 tag SNPs were predicted among the 99 associated SNPs with LD (D́ > 0.8) (Fig. 7). Majority haplotype blocks contained two SNPs and only two blocks contain 4, and 6 markers. The haplotype-traits association analysis showed the haplotype 2, 3, 4, 5, 9, and 12 associated with birth weight (Table 6), while four haplotypes of them associated with daily weight gain. The haplotype 8 containing six SNPs didn’t associate with body weight, while The haplotype 4 was the most important haplotype associated with body weight (Table 6).

**Table 5:** List of SNP markers associated with camel growth at −Log p value ≥  2.5.

SNP ID(Chr_pos)	SNP ID(Chr_pos)	SNP ID(Chr_pos)	SNP ID(Chr_pos)
S1_63040824	S10_13608620	S14_31079196	S19_10894231
S1_63040834	S10_13608628	S14_31241609	S19_11030167
S1_63048213	S10_13608631	S14_31241643	S19_11148798
S1_63445934	S10_13608637	S14_31244620	S19_9631630
S2_14965	S10_13608647	S14_31359918	S21_31508895
S2_99932513	S10_13608655	S14_31371222	S25_262766
S4_40844257	S10_13892353	S14_31371235	S25_262898
S6_14654249	S11_70990227	S14_31371236	S25_263079
S6_24997291	S11_71992855	S14_31371259	S25_263086
S7_75108807	S11_72060113	S16_34358452	S29_10601495
S7_82468596	S11_72060113	S16_34483240	S31_16977384
S7_82483675	S11_72350029	S17_75949	S31_16998649
S7_82483685	S11_72356390	S18_29830420	S33_4467956
S8_43104525	S11_72356400	S18_29958631	S34_6774561
S8_59919313	S11_72356401	S18_29963432	S34_6774561
S8_72594548	S11_72356436	S18_29963702	S35_6600815
S8_72594549	S11_74288613	S18_29985605	S35_9257022
S8_72594593	S11_74292760	S18_30061144	S35_9290500
S8_72594633	S11_81210420	S18_30075711	SX_113249264
S8_72594639	S12_5711	S18_30105059	SX_60452363
S9_15226704	S13_50711877	S19_10237641	SX_62116802
S9_22550930	S14_30854110	S19_10237661	SX_6887230
S9_22738164	S14_30865065	S19_10328831	SX_78141509
S9_53275637	S14_30865085	S19_10433012	SX_78141514
S9_53275650	S14_30865111	S19_10894226

**Table 6:** the haplotype-traits association analysis.

Trait	Haplotype	NSNP*	NSIG	ISIG	EMP1	SNPS
Birth Weight	Haplotype1	2	0	0	1	NaN**
	Haplotype2	2	2	1	1.00E-04	S8_72594549
	Haplotype3	2	2	1	0.0121	S9_53275650
	Haplotype4	6	4	1	0.0413	S10_13608620
	Haplotype5	2	2	1	0.0243	S11_72356401
	Haplotype6	2	0	0	1	NaN
	Haplotype7	2	0	0	1	NaN
	Haplotype8	4	0	0	1	NaN
	Haplotype9	2	2	1	4.00E-04	S19_10237661
	Haplotype10	2	0	0	1	NaN
	Haplotype11	2	0	0	1	NaN
	Haplotype12	2	2	1	0.009299	SX_78141514
Gain/day	Haplotype1	2	0	0	1	NaN
	Haplotype2	2	0	0	1	NaN
	Haplotype3	2	0	0	1	NaN
	Haplotype4	6	0	0	1	NaN
	Haplotype5	2	0	0	1	NaN
	Haplotype6	2	2	1	2.00E-04	S14_30865085
	Haplotype7	2	1	1	0.0391	S14_31241643
	Haplotype8	4	1	1	0.05859	S14_31371259
	Haplotype9	2	0	0	1	NaN
	Haplotype10	2	0	0	1	NaN
	Haplotype11	2	0	0	1	NaN
	Haplotype12	2	2	1	0.0011	SX_78141514
Body weight	Haplotype1	2	0	0	1	NaN
	Haplotype2	2	2	1	1.00E-04	S8_72594549
	Haplotype3	2	2	1	0.0128	S9_53275637
	Haplotype4	6	0	0	1	NaN
	Haplotype5	2	2	1	0.007299	S11_72356401
	Haplotype6	2	2	1	0.006499	S14_30865111
	Haplotype7	2	2	1	0.0203	S14_31241643
	Haplotype8	4	4	1	0.0395	S14_31371259
	Haplotype9	2	2	1	0.0034	S19_10237661
	Haplotype10	2	0	0	1	NaN
	Haplotype11	2	2	1	0.005999	S25_263086
	Haplotype12	2	2	1	0.038	SX_78141514

* NSNP: Number of SNPs in Haplotype, NSIG: Total number of SNPs below p-value threshold (p < 0.05)), ISIG: Number of significant SNPs also passing LD-criterion (R-squared > 0.50), EMP1: Empirical set-based p-value, SNPS: List of SNPs in the Haplotype.

** Not a Number.

### Genomic Selection Based on BGLR

**Figure 8:**
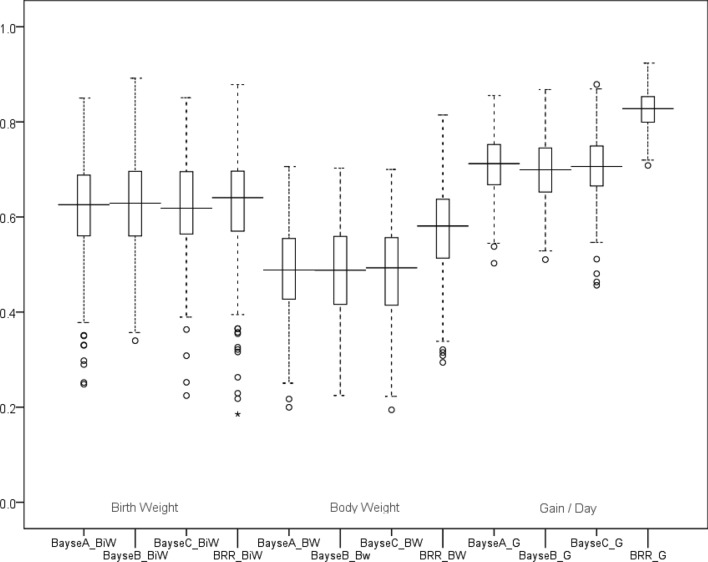
The accuracy of predicted GEBVs from the 99 SNPs using BGLR package.

Based on BRR model in BGLR, the GEBVs of each trait (birth weight, daily gain, and body weight) was estimated using all 14,522 SNPs. The GEBVs also were predicted using the 99 associated SNPs based on BRR, Bayes A, B, and C models. The averaged correlation (r) and regression (b) coefficient between the observed and the GEBVs predicted from two SNPs sets: (1) all 14,522 SNPs and (2) the 99 associated SNPs using BGLR package (BRR model). The accuracy of GEBVs were more than 0.65 base on all 14,522 SNPs, but the regression coefficients for birth weight, daily gain, and body weight were 0.39, 0.20, and 0.23, respectively (Table 7). the GEBVs was less biased based on the 99 associated SNPs. The accuracy of using the 99 associated SNPs also evaluated by cross- validation (3 folds and 100 replications) (Table 8). The accuracy of the BRR model was more than Bayes A, B, and C (r > 0.65) based on the 99 associated SNPs. the accuracy of GEBVs of body weight was less than birth weight and daily gain based on the 99 associated SNPs (Fig. 8).

**Table 7:** The averaged correlation (r) and regression coefficient (b) between the observed values and the GEBVs predicted from two SNPs sets: (1) all 14,522 SNPs and (2) the 99 associated SNPs using BGLR package (BRR model).

Trait	All 14,522 SNPs		99 Associated SNPs	
	r	b	r	b
Birth weight	0.96 ± 0.0004	0.39 ± 0.011	0.85 ± 0.0003	0.55 ± 0.0007
Daily gain	0.66 ± 0.002	0.20 ± 0.009	0.56 ± 0.0008	0.28 ± 0.0005
Body weight	0.86 ± 0.0009	0.23 ± 0.009	0.64 ± 0.0009	0.19 ± 0.0004

**Table 8:** The GEBVs accuracy predicted from the 99 SNPs using BGLR package.

	Model	r	SD	RMSE	R^2^
Birth weight	BRR	0.62	0.109	7.78	0.37
	Bayes A	0.61	0.103	7.876637	0.35
	Bayes B	0.62	0.101	7.826124	0.36
	Bayes C	0.62	0.109	7.826	0.36
Daily gain	BRR	0.82	0.069	69.064	0.67
	Bayes A	0.70	0.061	87.20	0.47
	Bayes B	0.69	0.067	88.12	0.46
	Bayes C	0.70	0.068	87.036	0.47
Body weight	BRR	0.57	0.094	37.66672	0.29
	Bayes A	0.48	0.096	40.3208	0.20
	Bayes B	0.48	0.099	40.31	0.20
	Bayes C	0.48	0.100	40.41	0.20

## Discussion

In this project, we performed a GWAS for growth traits in dromedary camels using the genotypes of 96 calves of the central desert of Iran. this is the first GWAS for birth weight, daily gain, and body weight of camels using GBS. The final goal of genetic mapping is to identify associated markers with phenotype^37,38^. A number of 14,522 SNPs, generated using GBS, prepared the GWAS in dromedary camels. We identified 36 candidate genes associated with camel’s growth using Annotation of the genomic region (s) within ± 100 kb of the associated 28 SNPs. The candidate genes for birth weight of camels are *ACTR3B, RPL32, XRCC2, SERAC1, TBX15, RNF114, IFNL1, SPATA2, SNAI1.* For daily gain trait, *EFCAB5, NSRP1, Slc6a4, ITGA7, OR6C2, RIPOR3, PTPN1, PARD6B, BCAS4, MOCS3, TRAPPC9* as candidate genes were identified. *EMX2, FAM184A, MCM9, ASF1, CSE1L, ARFGEF2, TSR2, WNK3, LPAR4, RTL3, PEG10, PBDC1, TRAPPC13, UNKNOWN, DEXI, TSPYL4, CIITA* were for camel body weight. The QTL regions and the genes: *ADAMTSL3, CAPN2, CAPN2, FABP6, ZEB2* were detected using GWAS in Colombian Brahman cattle that influencing growth and weight traits^39^. Lu et al.^11^ (2020) reported *SLCO2A1, LY6K, RALYL, AADACL3, C17H4orf45, BICC1, SHROOM2* as candidate genes for birth weight in Chinese Fine-Wool Sheep^11^. The candidate genes for Dry Matter Intake, Average Daily Gain, and Metabolic Body Weight based on the imputed 7.8 M WGS in chattels were reported: *SNORA70, B3GALT1, DDR2, GPR37, SYT1, LYZL1, RGS2, F13A1, SNORA31, LCORL, DPH6, PARD3, MOS, CRB1, CUL1, CCND2, ARRDC3, PLAG1, STC2, CARD11, TMEM72, SCGB1A1, ERICH6, ARRDC3, GALNT14, PLAG1, ERGIC1, AP3S2, A1CF*^40^. An, B. et al. (2020)^41^ identified the candidate genes for growth traits in Simmental beef cattle, including *SOX2, SNRPD1, RASGEF1B, EFNA5, PTBP1, SNX9, SV2C, PKDCC, SYNDIG1, AKR1E2, PRIM2, SLC37A1, LAP3, PCDH7, MANEA, LHCGR, P2RY1, MPZL1, LINGO2, CMIP,* and *WSCD1*. *LOC101903200, PARP4, GPA33, NADK, PREX2, FRMD4B* were identified as candidate gene for carcass weight in commercial Hanwoo cattle^42^.

Performance of genomic selection was determined by the prediction accuracy^43–45^. Until now, Genomic prediction has been conducted in many animal species. The accuracy of GEBVs for economic traits in beef cattle was predicted range 0.38 to 0.40^46,47^. Also, ranged from 0.18 to 0.33 for growth traits in New Zealand sheep breeds^48^ It was reported range from 0.40 to 0.50 for important traits in pig^49^.

The statistical model, marker density, LD, and sample size influenced on selection accuracy ^43^. The accuracy of GEBVs for growth traits was reported 0.391 (GBLUP) and 0.379 (Bayes Lasso) in Yak^50^. Because of low samples size in this research, it was suggested to predict unbiased GEBVs using the associated SNPs from GWAS. The accuracy of GEBVs of birth weight, daily gain, and body weight based on the 99 associated SNPs was 0.62, 0.82, and 0.57, respectively. Using the most significant associated SNPs, the reduced SNP panels were developed for many traits^51^. It was resulted by the Bayes models, that some fraction of the SNPs has zero effect on the trait^51^. The beef industry has been focused on collections of informative SNPs for subsets of traits that have the most economic effect and market opportunity^51^. The 600 SNPs (20 markers / chromosome) in Bovine relatively had the same predictive ability rather than 50 K SNP^52^, and 90% of 50 k SNPs had zero effect^52^. Garrick et al. 2011 reported that using only 384 SNPs by low costing can be achieved predictive ability for interest traits, so that the accuracies were range from 0.30 to 0.60 for growth, meat quality, and carcass weight^53^. The accuracies were predicted 0.28, 0.29, 0.39, and 0.43 using 50, 100, 150 or 200 SNPs for marbling in Angus^52^. the haplotype-traits association analysis may also provide additional power^54^.

Phenotyping especially pedigree data is now the principal limitation in camel breeding. Genomic predictions do not reliant on pedigree data^55^, therefore it can be suggested in camel breeding because of changing into intensive farming. Establishing of training populations across countries provides an opportunity to increase training data size and genomic data in dromedaries.

## Conclusion

Body weight is a critical economic trait for camels and it is necessary to plan the breeding program. Detection of the genomic regions associated with growth is important for MAS (marker assisted selection) or GS (genomic selection). This is the first genome-wide association study using GBS on dromedary camels, and identifies markers associated with growth traits. This could help to plan breeding programs for genetic improvement in dromedary camels. Further studies using a larger sample size and collaboration of stakeholders will allow confirmation of the associated SNPs and candidate genes identified in this project. Because of long generation interval, no artificial insemination, and seasonal reproduction, genomic selection based on the 99 SNPs associated with growth trait could be a first step into the direction of genetic improvement for body weight in Iranian dromedaries.
